# Genetic diversity of mitochondrial DNA D-loop sequences in UAE native chickens

**DOI:** 10.1016/j.psj.2026.106748

**Published:** 2026-03-08

**Authors:** A.R.K. Kullan, E.G. Neumann, O. Alsheblak, A.J. Noura

**Affiliations:** Department of Integrative Agriculture, United Arab Emirates University, P.O. Box 15551, Abu Dhabi, United Arab Emirates

**Keywords:** mtDNA D-loop, UAE indigenous chickens, haplogroups, genetic diversity and UAE

## Abstract

This study was conducted to access the genetic diversity and phylogenetic relationship of United Arab Emirates (UAE) native chickens to support conservation of their genetic resources. A total of 161 native chickens were examined for genetic diversity and phylogenetic analysis using partial mitochondrial d-loop sequences (758 bp). The observed haplotype and nucleotide diversities were 0.670 and 0.0028 respectively, A total of 31 polymorphic site and 20 haplotypes were identified. These haplotypes were classified into four distinct haplogroups (A, B, C, and E), with E (95.6%) being the prominent maternal lineages of UAE native chickens. Phylogenetic analysis revealed that the haplogroup E of UAE native chickens clustered with the Indian Red Junglefowl (Gallus gallus murghi), suggesting a South Asian maternal origin. This lineage may have arrived in the country via an ancient trading network, especially through the maritime routes of the Indian Ocean. Other haplogroups were associated with East Asia and Southeast Asia origin. These results suggest that UAE native chickens have multiple maternal origins. The genetic diversity identified in this research will aid in the conservation and breeding of these chickens, focusing on economically advantageous traits.

## Introduction

The domestic chicken (*Gallus gallus domesticus*) is among the most economically important and widely distributed livestock species globally (McGowan and Kirwan, 2020). Domestic chickens belong to genus Gallus. Their origins can be traced back to the domestication of the red junglefowl (*Gallus gallus* species) in South Asia and Southeast Asia, as well as the Ceylon junglefowl (*Gallus lafayettii*) in Sri Lanka and the green junglefowl (*Gallus varius*) in the Indonesian Islands, the grey junglefowl (*Gallus sonneratii*) in South India. From these origins, domestic chickens eventually spread to various regions around the world (Tixier-Boichard et al., 2011; Xiang et al., 2014; Liu et al., 2006; Kanginakudru et al., 2008).

The Indigenous chickens, also known as local or native chickens, are commonly raised in rural and backyard environments (Manyelo et al., 2020). They play a significant role in the livestock of small-scale farmers and serve as a reliable source of meat. These chickens are economically viable, culturally important. They are well-adapted to various environmental conditions, resistant to disease and can thrive with minimal feed and care (Bettridge et al., 2018). Understanding of genetic diversity, evolutionary origin, and population structure is important for increasing the productivity and conservation program of these chickens.

Mitochondrial DNA (mtDNA) has been widely utilized in evolutionary and population genetic research both among and within species, owing to its high mutation rate, substantial copy number, absence of recombination, and maternal inheritance (Harpending et al., 1998; Harrison, 1989; Galtier et al., 2009). In mtDNA, coding regions such as cytochrome b, ribosomal 12S RNA, 16S RNA, and the non-coding d-loop region have been extensively used for the genetic characterization of native chickens and for tracing their lineages (Pham et al., 2023). Of these, d-loop region serves as a highly informative genetic marker due to high rate of evolution, high mutation rate and limited repair mechanism (Muchadeyi et al., 2008). The d-loop region has been effectively utilized to analyze both intraspecific and interspecific relationships, trace maternal origins, and conduct phylogenetic studies in native chickens (Godinez et al., 2019; Islam et al., 2019; Miao et al., 2013; Osman et al., 2016; Teinlek et al., 2018). Mitochondrial DNA d-loop sequences have been used to demonstrate the diversity of both wild ancestors and domesticated breeds. Phylogenetic analysis utilizing d-loop sequences has indicated that the red junglefowl (*Gallus gallus*) is the primary wild ancestor of the domestic chicken (Liu et al., 2006; Mia et al., 2013; Mwacharo et al., 2011; Oka et al., 2007; Fulmihito et al., 1994, 1996). Liu et al. (2006), using d-loop sequences, proposed that modern day chickens have descended from various maternal lineages that originated from subspecies of the red jungle fowl located in South and Southeast Asia, along with southwest China. The author classified indigenous chickens into nine distinct haplogroups (A-I). Miao et al. (2013) used complete mitochondrial DNA to expand our understanding of the maternal history of chickens and defined 13 haplogroup (A-I, and W-Z). Later Huang et al. (2018) identified haplogroup V. These studies have shown that haplogroups A–G were common and were shared by domestic chickens and red jungle fowl (RJF), indicating multiple local domestication events in South Asia, Southwest China, and Southeast Asia (Liu et al., 2006; Miao et al., 2013).

**Fig. 1:**
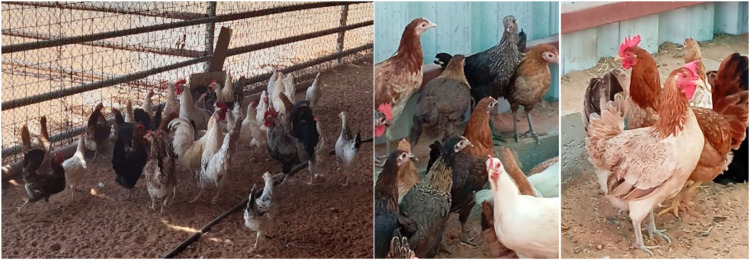
UAE local chickens used in this study, Local chickens of the UAE predominantly had multicolored plumage pattern, normal feather distribution, a single comb, white spotted red ear lobes and varying colors of shank and beaks.

Indigenous chickens in the United Arab Emirates (UAE) are commonly referred to as “Mahali chicken” or “Emirati local chicken”. The term “Mahali,” derived from Arabic, translates to “local,” reflecting their long-standing adaptation to the local environment and traditional backyard production systems. These chickens are generally small in body size and are predominantly found in the Al Ain region of UAE (Fig. 1). They exhibit huge phenotypic variations in terms of plumage color, feather morphology, shank color, comb type, ear lobe color and beak color (Tabbaa and Hassanin, 2018, Kullan et al. unpublished). These local chickens expected to possess the majority of the genetic diversity. To date no information is available on the genetic diversity and origin of UAE native chickens. In the present study, a partial d-loop segment (758 bp) of mtDNA was used to analyze the genetic diversity and evolutionary aspects of UAE native chickens.

## Material and methods

### Study material

The protocol for this experimental design was approved by the Animal Research Ethics Committee (A-REC, Approval Number – ERA_2024_5161) at United Arab Emirates University (UAEU). The research was carried out in the Al Ain region of the Emirate of Abu Dhabi, situated at coordinates 24° 12′ N to 27″ and 55° 44′ E to 41″, with an elevation of 252 meters above sea level. Native chickens used in this study were collected from Al Ain. A total of 161 individuals representing 12 households, each raising over 30 chickens were selected, and 10 to 15 individuals per farm were utilized in the current study. The blood samples were collected from the wing veins using a disposable syringe, placed in EDTA vials, and stored at 4°C until the DNA extraction process.

### DNA extraction, PCR amplification and sequencing

Genomic DNA was extracted from blood samples utilizing a commercial QIAamp DNA blood mini kit (Qiagen, Germany). The quality and quantity of the DNA samples were assessed using a spectrophotometer (Nano-Drop2000, Delaware USA), based on absorbance measurements at 260 and 280 nm, as well as through agarose gel electrophoresis analysis. The primer sequences for PCR amplification of d-loop region were: forward 5′ AGGACTACGGCTTGAAAAGC-3′ length and reverse-5′CCATACACGCAAACCGTCTC-3′ (Mwacharo JM et al., 2011). PCR reactions were conducted in a total volume of 10μl, which contains 20 ng of genomic DNA, 5 pmol of both forward and reverse primers, 1X PCR master mix (GeneDirex), and nuclease-free water. Amplification of the PCR reaction was carried out under the following cycling conditions: initial denaturation at 94°C for 5 min, followed by 35 cycles of denaturation at 94°C for 45 s, annealing at 55°C for 30 s, and extension at 72°C for 45 s, followed by a final extension at 72°C for 7 min. The resulting PCR product was subjected to electrophoresis on a 1% agarose gel and visualized using ultraviolet light. The PCR products were subsequently purified and sequenced using the Sanger method with a Biosystems 3500 Analyzer.

### Sequence analysis

Forward and reverse DNA sequence alignment, manual editing and assembly were performed using Bioedit v.7.2.5 (Hall, 1999). The alignment was refined to establish a nucleotide base pair of 758 bp. A Multiple Sequence Alignment was executed using the MUSCLE algorithm in MEGA v11 (Tamura et al., 2021). DNA sequence polymorphism, nucleotide diversity, and haplotype diversity were evaluated utilizing DnaSP v6.12.05 (Rozas et al., 2017). The MitoToolPy software (http://www.mitotool.org/) was used for haplogroup identification (Peng et al., 2015). Further to support the haplogroup affiliation of the UAE mahali chickens, 33 reference sequences were obtained from the gene bank and used to construct phylogenetic tree and MJ network (Table 1). Phylogenetic tree was generated using the neighbor-joining tree algorithm as implemented in MEGA v11 (Nei and Kumar, 2000; Tamura et al., 2021) and visualized by FigTree v1.4.4 (http://tree.bio.ed.ac.uk/software/figtree/). The evolutionary connections among the identified haplotypes were depicted through the construction of a median-joining network (Bandelt et al., 1999) using PopArt 1.7 (http://popart.otago.ac.nz). Tajima’s D neutrality test was performed to assess deviations from neutral evolution within the dominant haplogroup. The analysis was conducted using DnaSP v6.12.05 under default parameter (Rozas et al., 2017; Tajima, 1989). To illustrate the potential migratory routes and phylogenetic relationship of chickens in the Middle East, a total of 311 partial mitochondrial d-loop sequences (342 bp) from Saudi Arabia (185), Iran (107), Iraq (15 haplotypes representing 168 individuals), and Turkey (4 haplotypes representing 54 individuals) were retrieved from GenBank (Al-Jumaili et al., 2020; Meydan et al., 2016).

**Table 1:** Haplogroup names accession number and their respective breed and country used in this study.

S. No	Haplogroup	Genebank accession	Breed/Varieties	Country
1	A	GU261695.1	Red junglefowl (G. g. spadiceus)	China
2	A	GU261700	Gallus gallus	Myanmar
3	B	NC_007235.1	Red junglefowl (G. g. spadiceus)	Laos
4	B	GU261704.1	Red junglefowl (G. g. spadiceus)	Myanmar
5	C1	GU261701.1	Domestic chicken	China
6	C3	GU261707.1	Red junglefowl (G. g. murghi)	India
7	C3	GU261716.1	Red junglefowl (G. g. spadiceus)	Myanmar
8	D1	NC_007237.1	Red junglefowl (G. g. bankiva)	Indonesia
9	D1	NC_007236.1	Red junglefowl (G. g. gallus)	Philippine
10	D2	GU261683.1	Domestic chicken	China
11	E1	AP003317.1	White leghorn	Italy
12	E1	AY235570.1	New Hampshire Red	USA
13	E1	HQ857212.1	Domestic chicken	India
14	E3	GU261708.1	Red junglefowl (G. g. murghi)	India
15	E1	AY235571.1	New Hampshire Red	USA
16	E	GU261709.1	Red junglefowl (G. g. murghi)	India
17	E	HQ857210	Domestic chicken	India
18	E1	AP003318.1	White Plymouth Rock	England
19	F	GU261702.1	Red junglefowl (G. g. spadiceus)	China
20	F	GU261703.1	Red junglefowl (G. g. spadiceus)	Myanmar
21	G	GU261690.1	Red junglefowl (G. g. spadiceus)	China
22	H	AB268543.1	Gallus gallus	Japan
23	H	GU261715.1	Domestic chicken	China
24	I	GU261698.1	Domestic chicken	India
25	W	GU261706.1	Red junglefowl (G. g. spadiceus)	China
26	X	GU261692.1	Red junglefowl (G. g. spadiceus)	Chna
27	Y	GU261693.1	Red junglefowl (G. g. spadiceus)	China
28	Z	GU261674.1	Red junglefowl (G. g. jabouillei)	China
29	Z	GU261696	Red junglefowl	Chine
30	V	LC146461.1	Red junglefowl	Cambodia
31	V	LC146464.1	Red junglefowl	Cambodia
32		KP211418.1	Aseel	India
33		KP211421.1	Nicobari Black	India

## Results

### D-loop sequence variability and haplotype diversity in UAE Mahali chickens

A total of 31 variable sites were identified from the comparison of 161 partial mitochondrial d-loop sequences of UAE chickens. The sequence length for the 161 individuals was 758 bp. The nucleotide composition of this sequence comprised 23.5% A, 31.4% T, 29.8% C, and 15.3% G, resulting in a total of 54.9% *A* + *T* and 45.1% *G* + *C*. No insertion or deletion polymorphism was detected. Among the 31 polymorphic sites, 17 were found as singleton and the remaining sequences were identified as paraimony-informative polymorphic sites. A total of 28 transition and three transversion substitutions were observed. The polymorphic site at nucleotide positions 177, 193 and 256 were identified as transversion. Of the 31 polymorphic sites, 28 were located within the 167-446 bp regions (hypervariable region 1), while the remaining three were situated between the 686 to 711 bp region (Table 2).

**Table 2:** The haplotype identified in the mtDNA d-loop sequence of the Mahali chickens in the UAE.

	Nucleotide transition																																
Haplotype	1	1	1	2	2	2	2	2	2	2	2	2	2	2	2	2	3	3	3	3	3	3	3	3	3	3	4	4	6	6	7	Haplo groups	N
	6	7	9	1	1	1	2	2	4	4	4	5	6	6	8	9	0	1	1	1	2	3	4	5	6	9	1	4	8	9	1		
	7	7	3	0	2	7	2	5	3	6	9	6	1	5	1	3	6	0	3	5	2	0	2	5	3	1	7	6	6	7	1		
MH1	T	A	C	C	G	C	A	C	C	C	A	C	T	C	A	T	T	T	C	C	T	C	A	T	C	C	T	T	G	T	G	E	5
MH2	C	.	.	T	.	T	.	T	T	.	.	T	C	.	.	.	.	C	.	.	.	.	.	.	.	.	C	C	A	.	.	A	4
MH3	.	.	.	.	.	.	.	.	.	.	.	.	.	.	.	.	.	.	.	.	.	.	.	.	.	.	C	.	.	.	.	E	90
MH4	.	.	.	.	.	.	.	.	.	.	.	.	.	.	.	.	.	.	T	.	.	.	G	.	.	.	C	.	.	.	.	E	7
MH5	.	.	.	.	.	.	.	.	.	.	.	A	.	.	.	.	.	.	.	.	.	.	.	.	.	.	C	.	.	.	.	E1b	10
MH 6	.	.	.	.	.	.	.	.	T	.	.	.	.	.	G	.	.	.	.	.	.	.	.	.	.	.	C	.	.	.	.	E1b	16
MH 7	.	.	.	.	.	.	.	.	.	.	.	.	.	.	.	C	.	.	.	.	.	.	.	.	.	.	C	.	.	.	.	E	1
MH8	.	.	.	.	.	.	.	.	.	.	.	.	.	.	.	.	.	.	.	.	.	.	.	.	.	.	C	.	.	.	A	E	1
MH9	.	.	A	.	.	.	.	.	.	.	.	.	.	.	.	.	.	.	.	.	.	.	.	.	.	.	C	.	.	.	.	E	8
MH10	.	.	.	.	.	.	G	.	.	.	G	.	.	.	G	.	.	.	.	.	.	.	.	C	.	.	C	.	A	.	.	E3a	4
MH11	.	.	.	.	.	.	G	.	.	.	.	.	.	.	.	.	.	.	.	.	.	T	.	.	.	.	C	.	.	.	.	E1c	3
MH12	.	.	.	.	A	T	.	.	T	T	.	T	C	.	.	.	.	C	.	T	.	.	.	.	.	.	C	C	A	.	.	B1a	2
MH13	.	.	.	.	.	.	.	.	.	.	.	.	.	.	.	.	.	.	.	.	C	.	.	.	.	.	C	.	.	.	.	E	1
MH14	.	.	.	.	.	.	.	.	.	.	.	.	.	.	.	.	.	.	.	.	.	T	.	.	.	.	C	.	.	.	.	E1c	1
MH15	.	.	.	.	.	.	.	.	.	.	.	.	C	.	.	.	.	.	.	.	.	.	.	.	.	.	C	.	.	.	.	E	1
MH16	.	.	.	.	.	.	.	.	.	.	.	.	.	.	.	.	.	.	.	.	.	.	G	.	.	.	C	.	A	.	.	E	3
MH17	.	.	.	.	.	.	.	.	.	.	G	.	.	.	G	.	.	.	.	.	.	.	.	C	.	.	C	.	A	.	.	E3a	1
MH18	.	.	.	.	.	.	.	.	.	.	.	.	.	.	.	.	.	.	.	.	.	.	.	.	.	.	C	.	.	C	.	E	1
MH19	.	T	.	.	.	T	.	.	.	.	.	.	.	T	G	.	C	.	.	.	.	.	G	.	T	.	C	C	A	.	.	C3	1
MH20	.	.	.	.	.	.	G	.	.	.	G	.	.	.	.	.	.	.	.	.	.	.	.	C	.	T	C	.	.	.	.	E3a	1
																																	161

A total of 20 haplotypes, named as MH1 to MH20 (MH, Mahali Haplotype), were identified from 31 polymorphic sites (Table 2). All haplotypes have been submitted to GenBank (accession numbers PX753570-PX753589). Among the 20 haplotypes, the MH3 haplotype was the most prevalent, comprising 55%, followed by MH_6 at 9.9% and MH_5 at 5.6%. The remaining haplotypes each accounted for less than 5%, with some represented by only a single individual. The genetic diversity analysis indicated a haplotype diversity of 0.670 ± 0.04 and a nucleotide diversity of 0.0028 ± 0.0004. Partial sequence analysis of Middle Eastern populations (342bp) further showed that a large proportion of samples from Saudi Arabia (49.7%), Iraq (54%), Turkey (87%), and Iran (80%) shared with MH3 haplotype. The high frequency of MH3 haplotype suggests that it likely represents the ancestral haplotype in the UAE and the Middle East region.

### Phylogenetic and network analysis

**Fig. 2:**
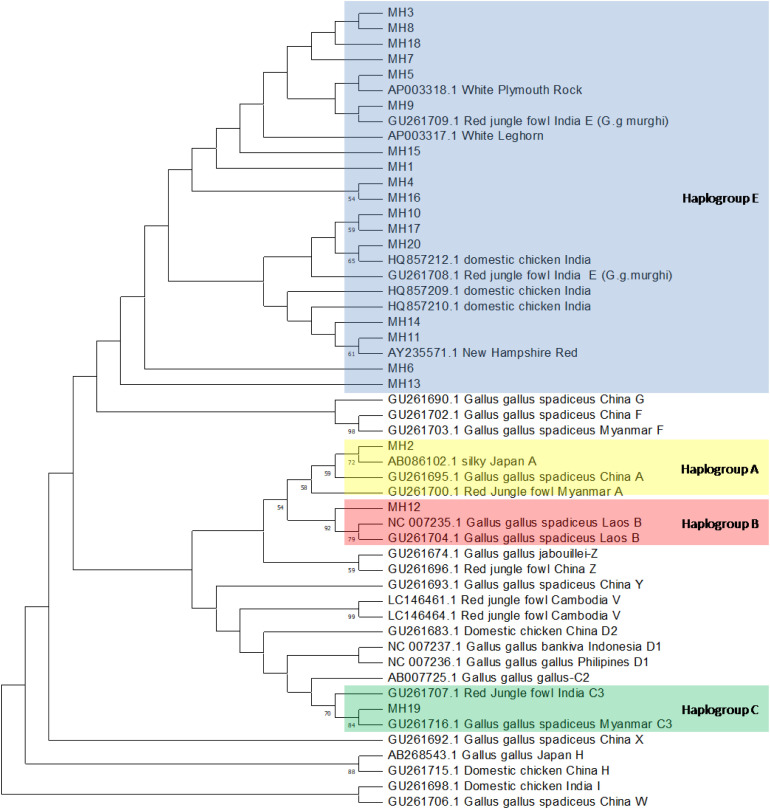
Neighnour-Joining phylogenetic tree for partial DNA sequences of UAE native chickens. A total of 20 haplotype of UAE native chickens and 33 reference sequences used for NJ tree construction. Node labels correspond to bootstrap values evaluated with 100 replicates. Bootstrap values under 50% are not shown.

Using the MitoToolPy program, a total of four haplogroups was determined for the UAE local chickens (A, B, C and E). The haplogroup E is the most predominant haplogroup found in the UAE chickens (E-73.3%, E1b- 16.1%, E1c −2.4%, E3a −3.5% of individuals) followed by haplogroup A (2.4%), B (B1a-1.2%) and C (C3-0.6%). Haplogroups A, B, and C were each represented by a single haplotype, with four, two, and one individuals respectively. The frequency of each haplogroup was mentioned in Table 3. Similarly phylogenic analysis using reference sequences clustered UAE native chickens haplotypes into the same four haplogroups (A, B, C and E, Fig. 2). Furthermore, the predominant haplogroup E clustered closely with the Indian Red Junglefowl (Gallus gallus murghi). Additionally, a phylogenetic analysis was conducted to evaluate the relationships among regional haplotypes using partial d-loop sequences from the Middle Eastern region (Saudi Arabia, Iraq, Iran and Turkey). The majority of haplotypes identified from the partial sequences of the Middle East were found to cluster with the reference sequence of haplogroup E (Fig. 4, Gallus gallus murghi).

**Table 3:** Haplogroups and their frequency identified in UAE native chickens.

S. No	Haplogroup	Number of individuals and Percentage
1	E	118 (73.3%)
2	E3a	6 (3.7%)
3	E1b	26 (16.1%)
4	E1c	4 (2.4%)
5	A	4 (2.4%)
6	B1a	2 (1.2%)
7	C3	1 (0.6%)
	Total	161

The median network analysis facilitates the identification of haplotype diversity and their interrelations. The median joining network was constructed using the 20 haplotype identified in this study in conjunction with 33 reference sequences that correspond to 14 haplogroups as defined by Miao et al. (2013). Similar to phylogenetic analysis, the haplotypes identified in this study were segregated into four distinct haplogroups (A, B, C and E). Among the twenty haplotypes, seventeen were designated to haplogroup E, while the remaining three were grouped to haplogroups A, B, and C.

**Fig. 3:**
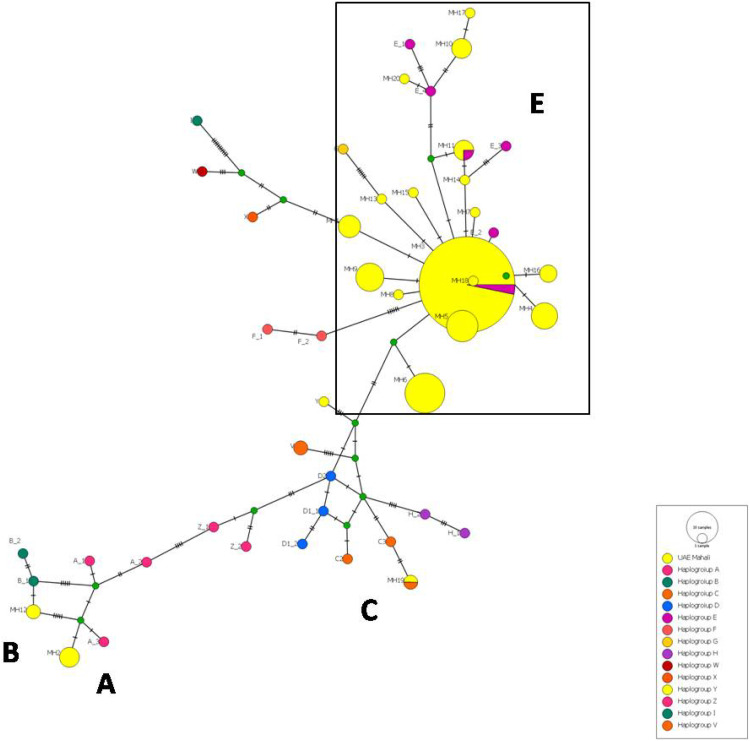
Median Joining (MJ) netwoodk of 20 haplotypes with 33 reference haplotypes based on the polymotphic sites of the partial mitochondrial d-loop region (758 bp). The circle sizes are corresponding to the haplotype frequencies. Different haplogroups were distinguished by the colors.

The predominant haplotype, MH3, is situated at the center in a star-like pattern (Fig. 3). Among the 14 haplogroups, only haplogroups F and G were distinguished from E by six mutations without median vectors, whereas the remaining haplogroups were differentiated from E by varying numbers of median vectors with mutations. Haplogroup E was separated from C by six mutation and D by three mutations with four and two median vectors respectively. Both A and B were distinguished by six mutations, whereas A differentiated from haplogroup D by twelve mutations. The median vectors may be attributed to unsampled haplotypes or haplotypes that have become extinct. The estimated Tajima’s D value for dominant haplogroup E was –1.52846, which was not statistically significant (*P* > 0.10).

**Fig. 4:**
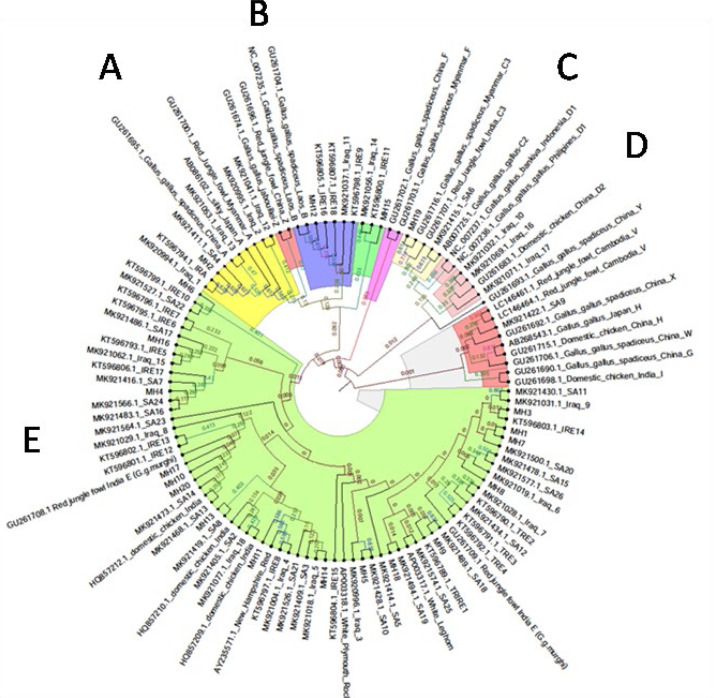
Neighbor-Joining phylogenetic tree constructed for partial DNA sequences (342 bp) from Middle Eastern samples. A total of 20 haplotypes from the UAE, 26 from Saudi Arabia, 18 from Iraq, 15 from Iran, and 4 from Turkey, along with 33 reference sequences, were utilized for the NJ tree construction. The majority of the haplotypes were clustered within haplogroup E.

## Discussion

The study of genetic diversity offers crucial insights for breeding initiatives aimed at enhancing genetic characteristics and operational efficiency (Guo et al., 2017). At present, there is a lack of information regarding genetic diversity studies and origin of indigenous chickens in the UAE. In the present study, partial d-loop sequence from 161 chickens from UAE native chickens was analyzed to dissect their haplotype and nucleotide diversity, mtDNA haplotypes, phylogenetic relationship and maternal ancestry. This is the first study reported on genetic diversity and origin of UAE native chickens.

The haplotype diversity and the nucleotide diversity were important factors in assessing genetic diversity In this study, partial (758 bp) mtDNA d-loop sequence from UAE Mahali chickens revealed 31 polymorphic sites resulted in 20 haplotype. The overall haplotype (0.670) and nucleotide diversity (0.002) identified in this study is somewhat lower compared to South Asia and Southeast Asian native chickens. Teinlek et al., 2018 reported 0.86 and 0.005 for Thai indigenous chickens, Godinez et al. reported 0.90 and 0.004 for Philippine native chickens, Cuc et al., 2011 reported 0.84 and 0.18 for Vietnamese native chickens, Gou et al. 2017 reported 0.92 and 0.012 for Chinease native chickens, Mon et al., 2021 reported 0.948 and 0.008 for Mynanmar native chickens and Nisar et al. (2019) reported 0.82 and 0,005 for Pakistan native chickens. In contrast, the native chickens of UAE showed somewhat higher haplotype and nucleotide diversity compared to certain African chicken population, namely the Sudaneas, Ugandan (Mwacharo et al., 2011), Nigerian (Makanjuolal et al., 2010; Tor et al., 2021) and South Africa (Mtileni et al., 2011). In the Middle East, Al-Qamashou et al. (2014) analyzed 175 village chickens from 11 sites across the Middle East and reported a haplotype diversity of 0.7588 and nucleotide diversity of 0.0065, while Al-Jumaili et al. (2020) similarly observed substantial diversity across five sites in Iraq (0.686, 0.0067) and 17 sites in Saudi Arabia (0.727, 0.0041). In contrast, Meydan et al. (2016) documented markedly lower diversity in Turkish (0.24, 0.0006) and moderate levels in Iranian chickens (0.36, 0.0021). The genetic diversity observed in the present study is comparable to, though slightly lower than, two Middle Eastern reports (Al-Qamashou et al., 2014; Al-Jumaili et al., 2020). This difference may reflect broader geographic sampling in previous studies, which likely captured slightly greater haplotypic variation.

Majority of the haplotypes (85%) identified in UAE Mahali chickens and Middle East haplotypes were clustered to haplogroup E and dominated with MH3 haplotype. This clustering pattern supports the predominance of haplogroup E lineages within Middle Eastern chicken populations and is consistent with previous reports (Al-Qamashou et al., 2014; Meydan et al., 2016; Al-Jumaili et al., 2020) and is believed to have originated from the Indian subcontinent (Kanginakudru et al., 2008; Miao et al., 2013). Haplotype E also become prevalent in commercial lines, as well as in European, Middle Eastern, African, Southeast Asian, and Indian native chickens (Liu et al., 2006; Miao et al., 2013; Godinez et al., 2021; Hata et al., 2021; Mwacharo et al., 2011). The occurrence of haplogroup E in the UAE and Middle East populations may reflect historical dispersal events facilitated by ancient maritime trade networks linking the Indian subcontinent with the Arabian Peninsula, particularly the coastal regions of present-day United Arab Emirates (UAE). Archaeological evidence indicates active maritime trade between Mesopotamia, the Arabian Gulf, and the Indus Valley Civilization as early as the third millennium BCE (Potts, 1990; Ray, 2003). Excavations in southeastern Arabia (e.g., UAE and Oman) have documented extensive trade contacts with South Asia, including the exchange of domesticated plants and animals (Beech, 2004; Fuller, 2011). Such long-standing maritime interactions could have contributed to the introduction and spread of Indian-derived chicken maternal lineages into the Middle East.

The negative Tajima’s D value suggests a trend toward an excess of low-frequency polymorphisms within haplogroup E, which may indicate population expansion or purifying selection (Hahn et al., 2002). In the present study, out of the 20 haplotypes, with the exception of three haplotypes (3, 5, and 6), all other haplotypes were represented by less than 5% of the individuals, and nine of the haplotypes were represented by only one individual. The abundance of low-frequency haplotype may indicates a deviation from equilibrium due to recent population expansion of UAE native chickens (Tajima, 1996). However, the result was not statistically significant (*P* > 0.10), indicating that the observed deviation from neutrality is not strong enough to conclusively infer demographic expansion or selection. Therefore, the haplogroup E population appears to be evolving largely under neutral expectations based on the present dataset.

Haplotypes A, B, and C were detected at low frequencies. These three haplotypes demonstrated a significant association with the East Asia and Southeast Asia regions, especially in China, where they were found to have a high frequency. Haplogroup C is less prevalent than haplogroups A and B, yet it remains widespread throughout Asia (Liu et al., 2006; Oka et al., 2007; Miao et al., 2013). Beyond Asia, these haplogroups were observed at lower frequencies, particularly in Africa, the Middle East, and Europe (Al-Jumaili et al., 2020; Liu et al., 2006; Mwacharo et al., 2011). In the Middle East, haplogroups A (12%) and B (12%) were reported in native chickens from Iran, Azerbaijan, and Turkmenistan (Liu et al., 2006) Likewise, haplogroup A (1%) was reported in Iranian native chickens, whereas haplogroups A (1%) and C (1%) were noted in native chickens from Saudi Arabia and Oman (Al-Qamashoui et al., 2014; Mydan et al., 2016; Al-Jumaili et al., 2020). Furthermore, Al-Jumaili et al. (2020) assigned 14% of the indigenous chickens in Iraq into haplogroup A. This study is the first to report all three haplogroups together (A, B, and C) in the Middle East, particularly in the UAE. The low frequency of these haplogroup found in the UAE and other Middle East regions, suggest their recent introduction through maritime trading network from South East Asia or recent introduction of improved commercial breeds (Dana et al., 2010).

## Conclusion

This study provides comprehensive insights into the genetic diversity, phylogenetic relationship and maternal origin of UAE native chickens. The results revealed that UAE native chickens dominated with haplogroup E, supporting a maternal origin linked to the Indian subcontinent. The presence of low frequent haplogroups (A, B and C) suggests possible historical gene flow from Southeast Asia or more recent introgression from commercial breeds. The UAE indigenous chickens have exhibit moderated genetic diversity, reflecting a relatively conserved but viable maternal genetic pool. Considering their economic importance, adaptability to arid environments, and potential genetic pool for future breeding programs, strategic conservation and sustainable management of this native genetic resource are strongly recommended

## Funding

This research is supported by ASPIRE, the technology program management pillar of Abu Dhabi’s Advanced Technology Research Council (ATRC), via the ASPIRE “The Virtrual Research Institutes Program”. Funding number—21R097.

## CRediT authorship contribution statement

**A.R.K. Kullan:** Writing – review & editing, Writing – original draft, Supervision, Formal analysis, Data curation, Conceptualization. **E.G. Neumann:** Writing – review & editing, Supervision, Funding acquisition, Conceptualization. **O. Alsheblak:** Supervision, Formal analysis, Data curation. **A.J. Noura:** Formal analysis, Data curation.

## Disclosures

The authors declare that they have no known competing financial interests or personal relationships that could have appeared to influence the work reported in this review.
